# Chromosome analysis of *Endochironomus albipennis* Meigen, 1830 and morphologically similar *Endochironomus* sp. (Diptera, Chironomidae) from water bodies of the Volga region, Russia

**DOI:** 10.3897/CompCytogen.v9i4.5172

**Published:** 2015-09-10

**Authors:** Natalya Durnova, Ludmila Sigareva, Olga Sinichkina

**Affiliations:** 1 Saratov State Medical University, Bolshaya Kazachya Street 112, Saratov 410012, Russia

**Keywords:** Diptera, Chironomidae, *Endochironomus albipennis*, *Endochironomus* sp., karyotype, polytene chromosomes, chromosomal polymorphism, Volga River

## Abstract

Based upon the detailed chromosome map of polytene chromosomes of the eurybiont species *Endochironomus albipennis* Meigen, 1830, the localization of the centromere regions using a C-banding technique is defined. Chromosomal polymorphism in populations from two water bodies in the Volga region has been studied, 17 sequences are described. Polytene chromosomes of *Endochironomus* sp. (2n=6), having larvae morphologically similar to those of *Endochironomus albipennis* Meigen, 1830 (2n=6) are described for the first time.

## Introduction

Larvae of *Endochironomus albipennis* Meigen, 1830 inhabit water bodies of different types. They are typical epibiotic organisms inhabiting submerged objects in the littoral zone, sometimes occurring also inside strongly decomposed plant residues (Kalugina 1963, Belyanina 1981). In Russia, this species is widely spread in the South and Center of the European part, in Siberia and in Kamchatka (Kalugina 1963, Belyanina 1981, Petrova and Michailova 1989).

The first data about the chromosome number of *Endochironomus albipennis* (2n=6) were reported by Konstantinov and Belyanina-Nesterova (1971). Later, a description of the karyotype and chromosomal polymorphism in a population from the Volga River was done by Belyanina (1981). This author indicated the chromosomes as: chromosome I (arms AB); chromosome II (arms CD); chromosome III (arms EF). Another description of chromosome arms including marking the chromosome regions was made by Michailova and Gercheva (1982) and Michailova (1987, 1989) for the Bulgarian and Swiss populations.

Kiknadze et al. (1991) mapped *Endochironomus albipennis* chromosomes using the photomap of Michailova (1987). Nevertheless designation of arms in chromosome III in their article does not conform to this system, i.e. numeration of parts (from 1 to 12) begins from the arm defined as F, whereas the same arm in photomap of Michailova (1987) is defined as arm GE. Arm GE in the photomap of Kiknadze et al. (1991) conforms to arm F in photomap of Michailova (1987). Chromosomal polymorphism of *Endochironomus albipennis* is still poorly studied, but several types of inversions have been described by Belyanina (1981) and Petrova and Michailova (1989).

There is neither a unified system of chromosome mapping nor a catalogue of chromosome sequences for *Endochironomus albipennis*. The few available photomaps are partially incomparable with each other. Therefore it is impossible to establish the limits of chromosome rearrangements in the populations of this species.

The main objectives of the present work were to study the chromosome polymorphism in two populations of *Endochironomus albipennis* from the Volga region and to present the list of chromosome sequences of the species. In addition, our aim was to provide the first description of polytene chromosomes of *Endochironomus* sp., larvae of which are similar in morphology to those of *Endochironomus albipennis*.

## Material and methods

The investigations were carried out in three stations in the Volga region (near Saratov). Sixty eight larvae of *Endochironomus albipennis* were collected in Sazanka Lake, Engels (51°29'52"N, 46°4'11"E) and in a pond near Novo-Aleksandrovka village (48°21'00"N, 31°29'00"E). Thirteen larvae of *Endochironomus* sp. were collected 11.08.2010 in Saratovka River (51°31'9"N, 46°15'57"E) inside decomposing rhizomes of *Nuphar luteum* (Linnaeus, 1753).

The species were identifying using larval morphology (Pankratova 1983, Pinder and Reiss 1983). The preparations of the polythene chromosomes were made from squashes of salivary glands cells stained with the ethanol-orcein method (Demin and Shobanov 1990). For detection of heterochromatin and centromere regions in chromosomes, a method of C-banding described by Belyanina and Sigareva (1978) was used.

**Figure 1.**
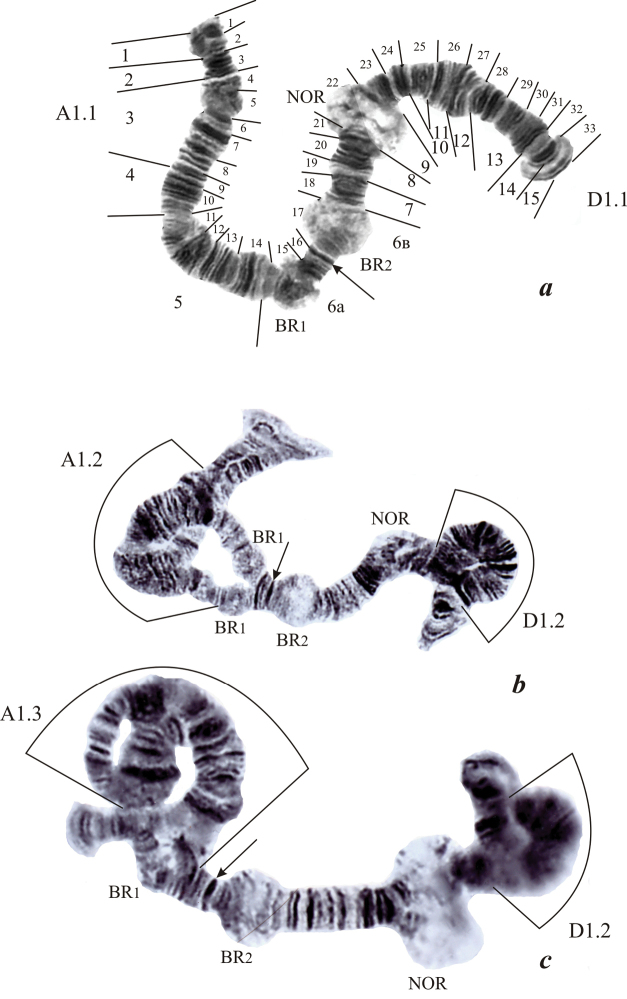
Chromosome I (AD) in the karyotype of *Endochironomus albipennis*: **a** homozygous for chromosomal sequences in the arms A (A1.1) and D (D1.1) **b** chromosome I (AD) with two heterozygous inversions – in the arm A (A1.2) and D (D1.2) **c** chromosome I with two heterozygous inversions – in the arm A (A1.3) and D (D1.2). Chromosome arms after Michailova (1987). The large regions of chromosome are presented according to Michailova (1987), small regions of chromosome done in this study were marked over the chromosome. The regions with inversions are marked by the brackets, Nucleolus Organizer (NOR), Balbiani ring (BR), puff (p), arrows indicates the centromere of the chromosome I (AD).

**Figure 2.**
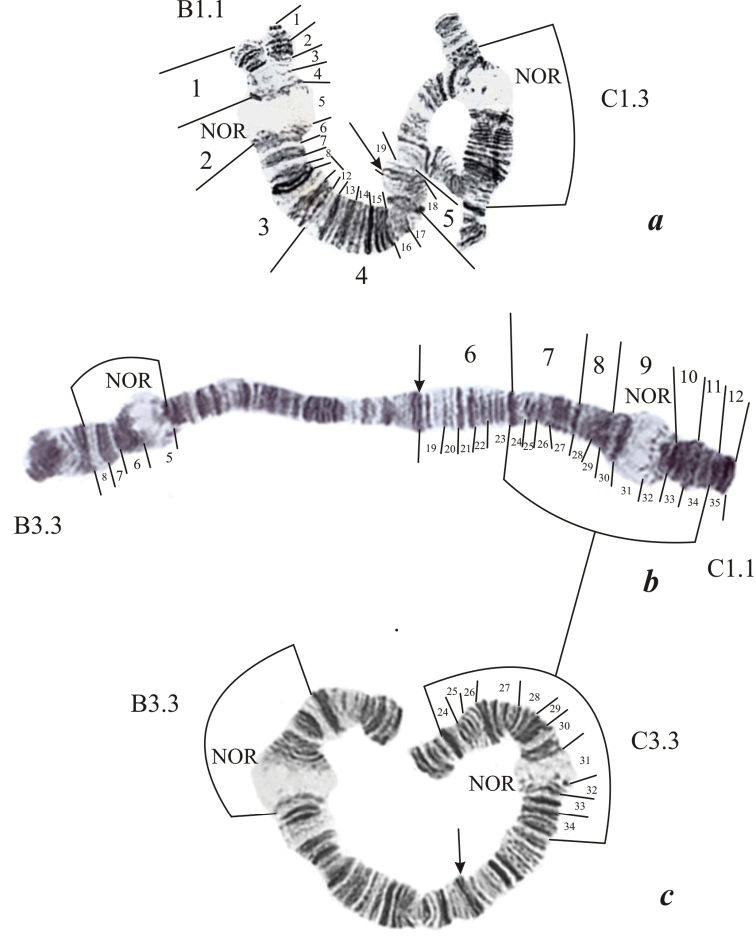
Chromosome II (BC) in the karyotype of *Endochironomus albipennis*: **a** homozygous for chromosomal sequences B1 in the arm B (B1.1) and heterozygous for the sequence C3 (C1.3) **b** chromosome II (BC), for B3 (B3.3) and C1 (C1.1) **c** chromosome II (BC) with two homozygous inversions (B3.3 and C3.3). The designations are the same as in Fig. 1.

**Figure 3.**
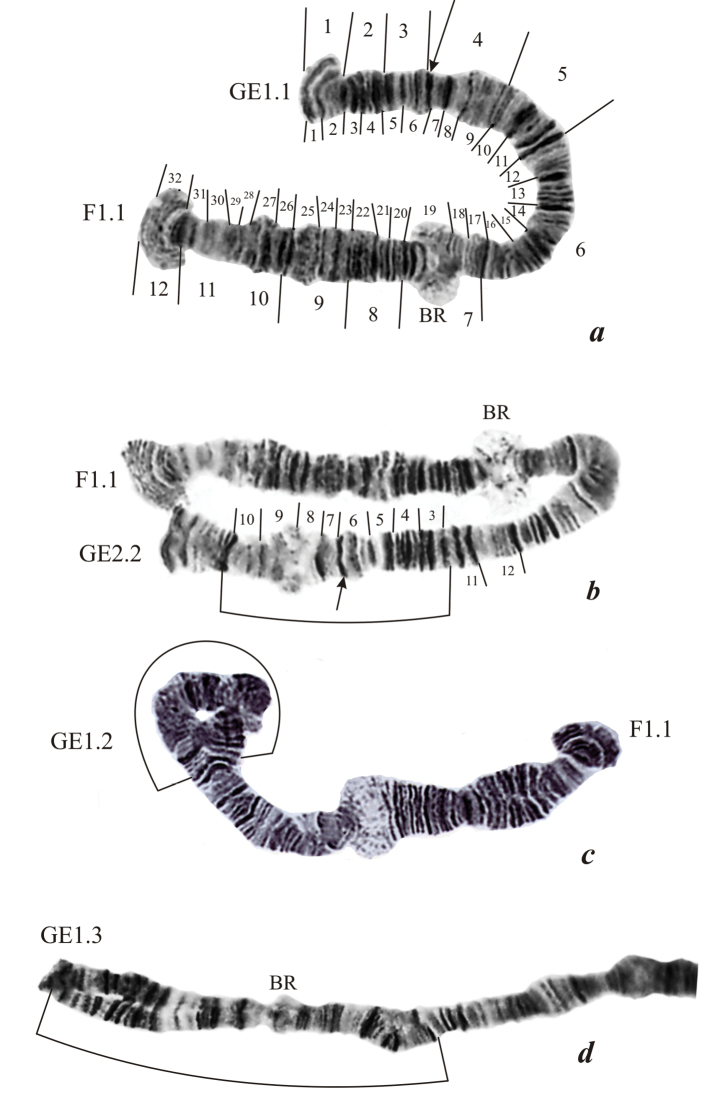
Chromosome III (GEF) in the karyotype of *Endochironomus albipennis*: **a** homozygous for chromosomal sequences in the arms GE (GE1.1) and in the arm F (F1.1) **b** homozygous inversion GE2.2 **c** heterozygous inversion GE1.2 **d** heterozygous inversion GE1.3. The designations are the same as in Fig. 1.

Designation of the polythene chromosome arms was made according to Michailova (1987). In the chromosome map of *Endochironomus albipennis* (Figs 1a, 2a, b, 3a) we have saved the marking of large regions (marked by large numerals at the pictures) conforming to the mapping system developed by Michailova and Gercheva (1982) and Michailova (1987, 1989). We developed here a more detailed mapping including the separation of the small regions (marked by small numerals under the chromosome) of chromosomes (Table 1, Figs 1–3).

**Table 1.** Chromosome arms and banding sequences in the polytene chromosomes of *Endochironomus albipennis*.

Chromosome arms (Belyanina 1981)	Chromosome arms (Michailova 1987, 1989)	Banding sequences (Michailova 1987)	Banding sequences (this study)
C	A	1–6a	*alb*A1 (1–16)
		-	*alb*A2 (inversion of section 4–14)
		-	*alb*A3 (inversion of section 4–15)
D	D	6b-15	*alb*D1 (17–33)
		inversion of section 10–13	*alb*D2 (inversion of section 24–31)
		-	*alb*D3 (inversion of section 22–31)
A	B	1–5	*alb*B1 (1–17)
		-	*alb*B2 (inversion of section 9–12?)
		-	*alb*B3 (inversion of section 5–8)
B	C	6–12	*alb*C1 (19–35)
		-	*alb*C2 (inversion of section 18–32)
		-	*alb*C3 (inversion of section 24–34)
E	GE	1–6	*alb*GE1 (1–16)
		-	*alb*GE2 (inversion of section 3–10)
		-	*alb*GE3 (some inversion steps)
F	F	7–12	*alb*F1 (17–32)
		inversion of section 8–9	*alb*F2 (inversion of section 21–25)

Designation of the band patterns conforms to the order of their description: *alb*A1, *alb*A2 etc. Genotypic combinations of banding sequences in every arm were designated as A1.1, A1.2, A2.2, etc., respectively. For analyzing chromosomal polymorphism we calculated the frequencies for every combination of chromosome sequences in each chromosome arm and also the mean number of heterozygous inversions per individual.

Analysis of slides was performed under the microscope MBI-11У4.2. For photomicrography a digital photographic camera Panasonic LS80 LUMIX was used. In the description of the larval morphology the terminology by Saether (1980) was used.

## Results

### *Endochironomus albipennis*. 2n=6. (Figs 1–4)

**Karyotype.** Centromeres are not distinct morphologically. Based on the C-banding patterns, chromosomes I (AD) and II (BC) are metacentric, whereas chromosome III (GEF) is acrocentric (Fig. 3). Chromosome arms designated previously by different authors are offered in Table 1. Frequencies (%) of chromosome inversions are presented in Table 2.

**Figure 4.**
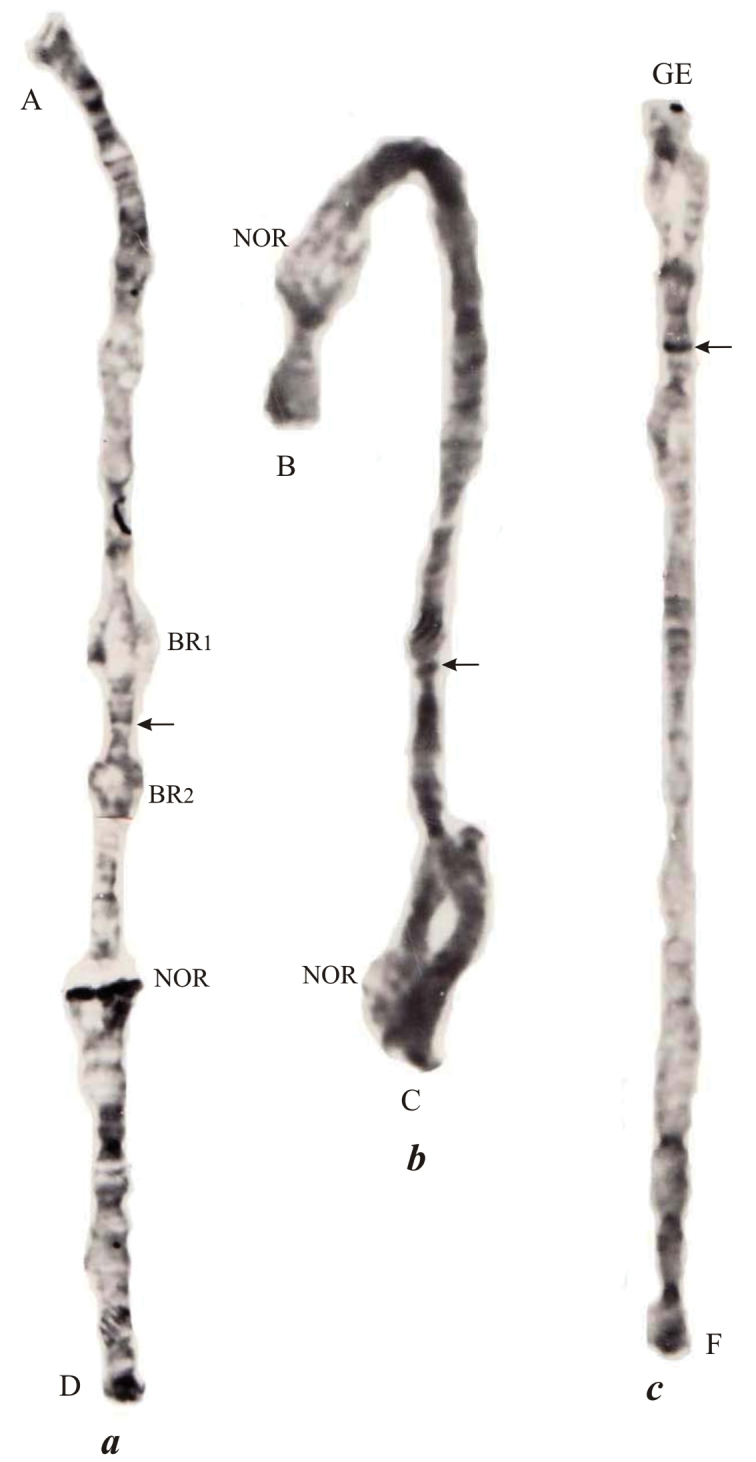
Localization of the centromere regions in the polythene chromosomes of *Endochironomus albipennis* by C band staining: **a** chromosome I (AD) **b** chromosome II (BC) **c** chromosome III (GEF). The designations are the same as in Fig. 1.

**Table 2.** Frequencies (%) of chromosome inversions in the polytene chromosomes of *Endochironomus albipennis*.

Genotypic combinations	Frequencies (%)		
	Sazanka Lake, Engels, 12.10.2008, 51 individuals (Present data)	Pond near Novo- Aleksandrovka village, 10.05.2009, 17 individuals (Present data)	Volga River, Saratov, 1971–1972, 1976–1979; 126 individuals (Belyanina 1981)
A1.1	22.7	52.9	39.7
A1.2	46.0	23.5	60.3 (C/C1)
A2.2	3.9	-	-
A1.3	27.4	23.5	-
D1.1	60.7	88.2	71.4
D1.2	23.5	5.8	28.6 (D/D1)
D2.2	1.9	5.8	-
D1.3	11.7	-	-
B1.1	37.2	29.4	85.7
B1.2	-	-	14.3 (A/A1)
B3.3	43.1	58.8	-
B1.3	17.6	11.7	-
C1.1	43.1	29.4	25.4
C1.2	-	-	74.6 (B/B1)
C1.3	23.5	17.6	-
C3.3	27.4	47.0	-
EG1.1	25.4	58.8	-
EG1.2	43.1	23.5	-
EG2.2	9.8	-	-
EG1.3	19.6	17.6	-
F1.1	98.1	100	-
F1.2	1.9	-	-
Number of heterozygous inversions per individual	1.6	1.3	3.2

Chromosome I (AD).The centromere region was detected using C-banding technique (Fig. 4b) as a thin indistinct C-band on the boundary of sections 16 and 17.

**Arm A** (Fig. 1a) has the following band sequence: 1 2 3 4 5 6 7 8 9 10 11 12 13 14 15 16 (Table 1, Fig. 1a). Balbiani Ring (BR1) is located in section 15. Section 5 contains a weakly active puff; on the boundary of sections 10 and 11 there is a constriction. Sequence *alb*A2 apparently was formed on sequence *alb*A1 as a result of inversion of sections 4-14. Sequence *alb*A2 was present both in homo- and heterozygous states (Fig. 1b). Homozygous inversion A2.2 was found for the first time; heterozygous inversion A1.2 was described previously as C/C1 by Belyanina (1981) and was observed with high frequency in larvae from the Volga River (Table 2). The chromosome sequence *alb*A3 found here for the first time arose apparently as a result of inversion of sections 4-15 (Fig. 1c) and occurred only in a heterozygous state – A1.3 (Table 2).

**Arm D** (Fig. 1a) has the band sequence: 17 18 19 20 21 22 23 24 25 26 27 28 29 30 31 32 33. In section 17, *BR_2_* is situated (Fig. 1a). The Nucleolus Organizer Region (NOR) is located in section 22 and shows a variable degree of activity. Sequence *alb*D2 was apparently formed on sequence *alb*D1 as a result of inversion of sections 24-31 and was present in the heterozygous state – D1.2 (Fig. 1b, c). The new sequence *alb*D3 was apparently formed on the sequence *alb*D1 as a result of inversion of sections 22-31. The homozygous inversion D2.2 and heterozygous inversion D1.3 were found for the first time, heterozygous inversion D1.2 was described previously as D/D1 by Belyanina (1981) and was also observed in larvae from Volga (Table 2).

Chromosome II (BC).The centromere region is detected using C-banding (Fig. 4 a) as an indistinct C-disc on the boundary between sections 18 and 19.

**Arm B** (Fig. 2a) has the band sequence: 1 2 3 4 5 6 7 8 9 10 11 12 13 14 15 16 17 18. The NOR is situated in section 5; no other active regions are present in this arm. For the first time we revealed the chromosome sequence *alb*B3, that had apparently arisen on sequence *alb*B1 as a result of inversion of sections 5-8 and was present both in the homozygous (Fig. 2b, c) and heterozygous states. The heterozygous inversion B1.2 was described previously as A/A1 by Belyanina (1981) and was not observed in our study (Table 2).

**Arm C** (Fig. 2b) has the band sequence: 19 20 21 22 23 24 25 26 27 28 29 30 31 32 33 34 35. The NOR is situated in section 31. Chromosome sequence *alb*C3 was formed as a result of inversion in sections 24-34 and was present in both states, heterozygous – C1.3 (Fig. 2a) or homozygous – C3.3 (Fig. 2c). The inversions C1.3 and C3.3 were found for the first time; the heterozygous inversion C1.2 was previously described as B/B1 by Belyanina (1981) and was observed only in larvae from the Volga (Table 2).

Chromosome III (GEF). Previously it was suggested that this chromosome is the result of tandem fusion of the short chromosome IV with arm E of chromosome EF but the division into arms «GE» and F was made without using C-staining (Michailova 1987). C-banding in this chromosome has clearly detected a C-disc (Fig. 4) on the boundary of sections 6 and 7. This C-positive disc is possibly the active centromere suggesting thus the chromosome III is heterobrachial with short arm G and long arm EF.

**Arm GE** (Fig. 3a) has the band sequence: 1 2 3 4 5 6 7 8 9 10 11 12 13 14 15 16. The active regions in this arm were absent. We have discovered here three chromosome sequences, among them *alb*GE1, accepted as a standard, and two sequences defined as *alb*GE2 and *alb*GE3 respectively. Sequence *alb*GE2 was formed as a result of inversion of sections 3-10 and found in both states, heterozygous GE1.2 (Fig. 3c) and homozygous GE2.2 (Fig. 3b). Sequence *alb*GE3 was found only in a heterozygous state (Fig. 3d); this is a complicated inversion formed through several inversion steps. The heterozygous inversions GE1.2 and GE1.3 were found in two reservoirs, whereas homozygous GE2.2 in the Sazanka Lake only (Table 2).

**Arm F** (Fig. 3a) has the band sequence: 17 18 19 20 21 22 23 24 25 26 27 28 29 30 31 32. *BR* is situated in section 19. We have found one inversion sequence defined as *alb*F2. Sequence *alb*F2 was formed on sequence *alb*F1 as a result of inversion of sections 21-25 and was present only in the heterozygous state. Frequency of this inversion was very low, 1.9%. It was only found in the population from the Sazanka Lake.

Analysis of chromosome polymorphism was performed in comparison with the data of Belyanina (1981) and Michailova (1987) (Table 2). A total of 17 chromosome sequences were recorded, which were found in the studied populations in homozygous and heterozygous states (Table 2).The level of *Endochironomus albipennis*`s chromosomal polymorphism in populations from different water bodies was essentially lower (number of heterozygous inversions per individual was 1.6 in Sazanka Lake, and 1.3 in pond near Novo-Aleksandrovka village), than in the Volga River near Saratov – 3.2 (Belyanina 1981).

### *Endochironomus* sp. 2n=6. (Figs 5, 6).

**Figure 5.**
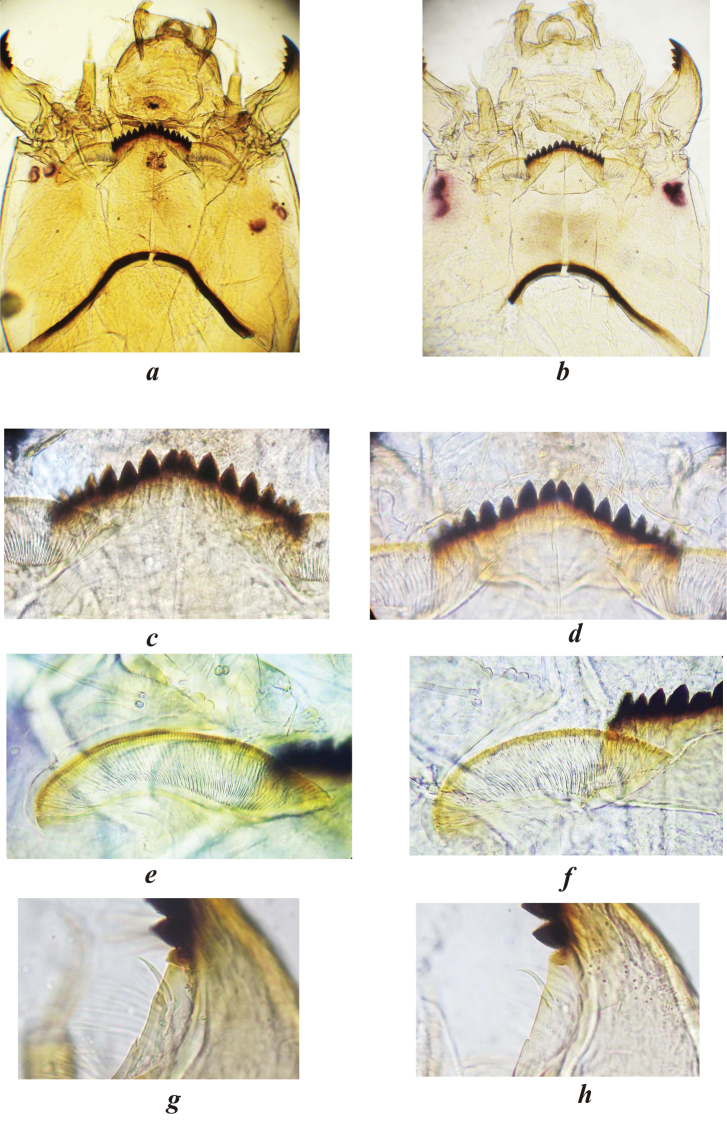
Larvae of *Endochironomus albipennis* (**a, c, e, g**) and *Endochironomus* sp. with 2n=6 (**b, d, f, h**): **a**, **b** head capsule (ventral view) **c**, **d** mentum **e**, **f** VmP (ventromental plates)
**g**, **h** SSd (seta subdentalis).

**Larva.** Body is yellow, maximal length - 10 mm. The head capsule is light yellow. Submentum of *Endochironomus* sp. (Fig. 5b) with a small pigment spot, as opposed to submentum of *Endochironomus albipennis* (Fig. 5a), which does not have a spot. Both species are similar in structure mental teeth (Fig. 5c, d), but differ significantly in structure of ventromental plates (VmP): VmP of *Endochironomus albipennis* (Fig. 5e) extend in width, the ratio of width to the length (VmPR) is 4.1–4.5 (4.2), VmP of *Endochironomus* sp. (Fig. 5f) less elongated in width, VmPR is 2.3–3.6 (3.0). Anterior edge of the ventromental plate of *Endochironomus albipennis* with a row of small, not protruding teeth, anterior edge of the VmP of *Endochironomus* sp. with a well-visible row of teeth. Seta subdentalis (SSd) of *Endochironomus albipennis* is lanceolate and straight (Fig. 5g), but SSd of *Endochironomus* sp. (Fig. 5h) is lanceolate and slightly curved.

**Karyotype.** Centromeres are not distinct morphologically. Chromosome arms were designated in accordance with the photomap of *Endochironomus albipennis*: I (AD), II (BC), III (GEF), I<II=III.

**Figure 6.**
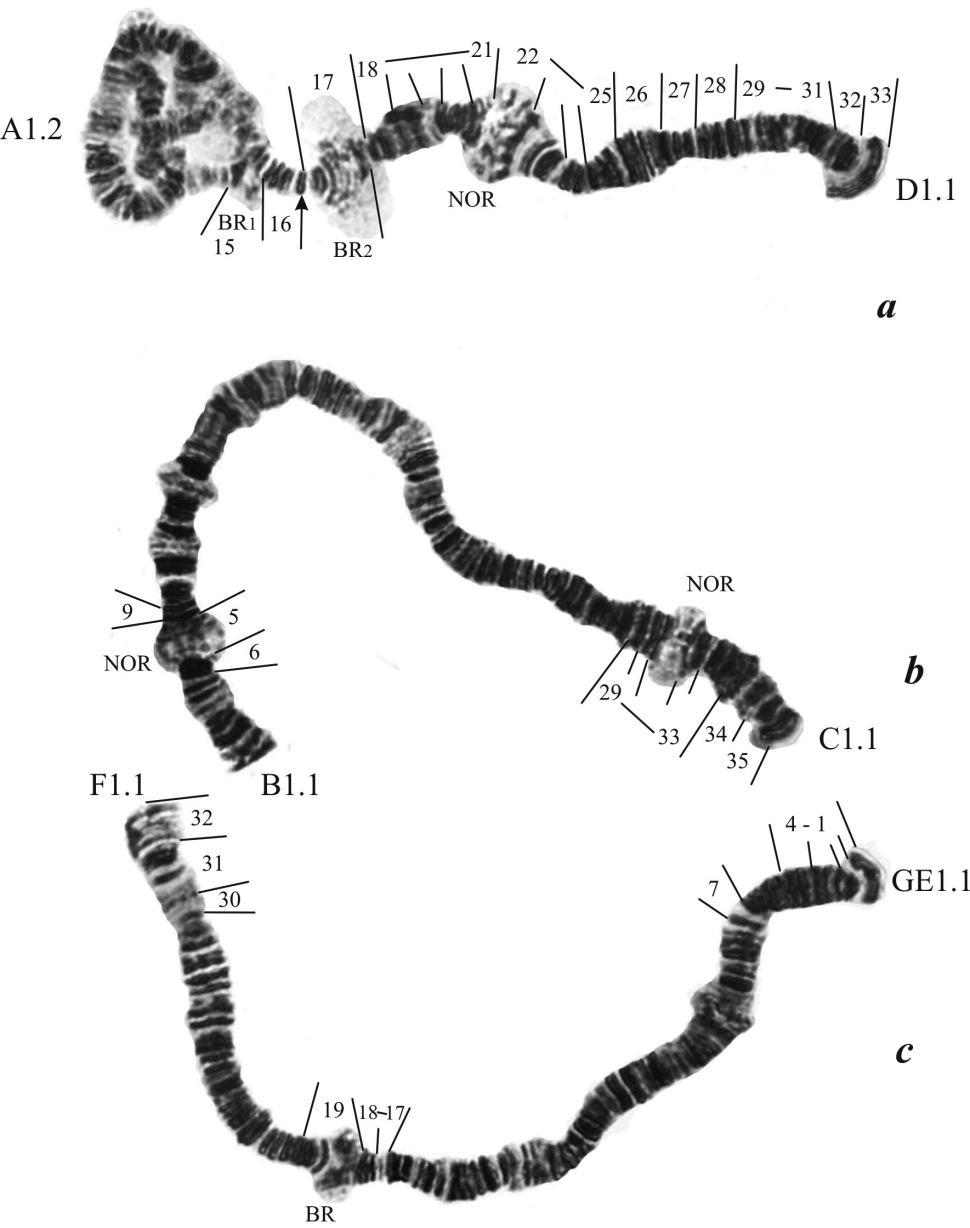
Karyotype of *Endochironomus* sp. (2n=6): **a** chromosome I (AD) **b** chromosome II (BC) **c** chromosome III (GEF). The designations are the same as in Fig. 1.

**Arm A** (Fig. 6a) has the region including sections 15-16 which is homeologous with arm A of *Endochironomus albipennis*. There is an active BR*_1_* in section 15.

**Arm D** (Fig. 6a) has the band sequence: 17 18 19 20 21 22 23 24 25 26 27 28 29 30 31 32 33. In section 17, BR*_2_* is localized, in section 22 one NOR is situated.

**Arm B** (Fig. 6b) has the small region including sections 5-6 which is homeologous with arm B of *Endochironomus albipennis*. In section 5, a NOR is situated.

**Arm C** (Fig. 6b) has the region including sections 29-35 which is homeologous with arm C of *Endochironomus albipennis*. In section 31, a NOR is situated.

**Arm GE** (Fig. 6c) has the only the region including sections 1-4 which is homeologous to arm GE of *Endochironomus albipennis*.

**Arm F** (Fig. 6c) has the sites including sections 17-19 and 30-32 which are homeologous with arm F of *Endochironomus albipennis*. In section 19, BR is situated.

## Discussion

Among all *Endochironomus* species detailed cytophotomaps of polytene chromosomes have been earlier compiled only for *Endochironomus tendens* Fabricius, 1775 (Durnova 2009), so a comparative analysis of polytene chromosomes of *Endochironomus tendens*, *Endochironomus albipennis* and *Endochironomus* sp. is currently hampered. Karyotypes of *Endochironomus tendens* and *Endochironomus albipennis* differ strongly both in disc patterns and in distinctness of centromere regions: in *Endochironomus tendens* centromere regions appear as thick heterochromatin blocks, whereas in *Endochironomus albipennis* they are morphologically not distinct. With the differential staining of chromosomes of *Endochironomus albipennis* using C-technique described by Sigareva (1985), centromeric heterochromatin was only defined clearly and permanently as a thin C-disc in the chromosome III (Fig. 4). Centromere regions of the chromosomes I and II were stained indistinctly, which is apparently connected with the very low amount of paracentromeric heterochromatin in these chromosomes.

The evolution of *Endochironomus tendens* apparently proceeded as a narrow specialization because larvae of this species are the typical miners in the tissues of littoral macrophytes (Kalugina 1963, Durnova 2009). Larvae of *Endochironomus albipennis* are eurybiontic and inhabit different biotopes being epibiotic organisms of different submerged littoral substrata in the water bodies. Molecular data (Durnova et al. 2014) have shown that by the nucleotide sequences of the mitochondrial gene *COI* *Endochironomus tendens* displays greater similarity to *Synendotendipes kaluginae* Durnova, 2010 than to *Endochironomus albipennis*, which indicates a high degree of divergence between *Endochironomus tendens* and *Endochironomus albipennis* not only at the chromosome level, but at the molecular level.

Larvae of *Endochironomus* sp. (2n=6) are morphologically similar to those of *Endochironomus albipennis* (Durnova et al. 2011). The degree of homeology in chromosome I (AD) between *Endochironomus albipennis* and *Endochironomus* sp. is relatively high; arms D are identical in banding patterns. These species differ in many sections of the chromosomes II (BC) and III (GEF), and only in few regions some common banding patterns can be seen. The number of discs in the central part of the chromosome II (BC) of *Endochironomus* sp., in which no homeology is observed, is much higher than in *Endochironomus albipennis*. Probably during a process of differentiation of these species, duplication of chromosome material took place. The degree of homeology between two species in chromosome I (GEF) is also low, length of arms F and GE of *Endochironomus* sp. exceeds considerably length of *alb*F and *alb*GE, which is probably related to the duplication of the chromosome material.

Thus, *Endochironomus* sp. distinctly differs from *Endochironomus albipennis* by the polytene chromosome band patterns, which undoubtedly argues for its separate species status. The chromosome differentiation of these two species was evidently accompanied not only by inversions, but also by duplications of chromosome material (in chromosome I and chromosome II), as indicated by larger number of discs in chromosomes of *Endochironomus* sp. as compared to *Endochironomus albipennis*.
